# Effects of Electromagnetic Fields on Osteogenesis of Human Alveolar Bone-Derived Mesenchymal Stem Cells

**DOI:** 10.1155/2013/296019

**Published:** 2013-06-19

**Authors:** KiTaek Lim, Jin Hexiu, Jangho Kim, Hoon Seonwoo, Woo Jae Cho, Pill-Hoon Choung, Jong Hoon Chung

**Affiliations:** ^1^Department of Biosystems & Biomaterials Science and Engineering, Seoul National University, Seoul 151-921, Republic of Korea; ^2^Department of Oral and Maxillofacial Surgery and Dental Research Institute, School of Dentistry, Seoul National University, Seoul 110-744, Republic of Korea; ^3^Department of Oral and Maxillofacial Surgery, Tooth Bioengineering National Research Lab, School of Dentistry, Seoul National University, Seoul 110-744, Republic of Korea; ^4^Tooth Bioengineering National Research Laboratory of Post BK21, School of Dentistry, Seoul National University, Seoul 110-744, Republic of Korea; ^5^Research Institute for Agriculture and Life Sciences, Seoul National University, Seoul 151-921, Republic of Korea

## Abstract

This study was performed to investigate the effects of extremely low frequency pulsed electromagnetic fields (ELF-PEMFs) on the proliferation and differentiation of human alveolar bone-derived mesenchymal stem cells (hABMSCs). Osteogenesis is a complex series of events involving the differentiation of mesenchymal stem cells to generate new bone. In this study, we examined not merely the effect of ELF-PEMFs on cell proliferation, alkaline phosphatase (ALP) activity, and mineralization of the extracellular matrix but vinculin, vimentin, and calmodulin (CaM) expressions in hABMSCs during osteogenic differentiation. Exposure of hABMSCs to ELF-PEMFs increased proliferation by 15% compared to untreated cells at day 5. In addition, exposure to ELF-PEMFs significantly increased ALP expression during the early stages of osteogenesis and substantially enhanced mineralization near the midpoint of osteogenesis within 2 weeks. ELF-PEMFs also increased vinculin, vimentin, and CaM expressions, compared to control. In particular, CaM indicated that ELF-PEMFs significantly altered the expression of osteogenesis-related genes. The results indicated that ELF-PEMFs could enhance early cell proliferation in hABMSCs-mediated osteogenesis and accelerate the osteogenesis.

## 1. Introduction

We established an *in vitro* cell stimulation culture that was based on extremely low frequency pulsed electromagnetic fields (ELF-PEMFs) which were performed to find out the effects on the proliferation and differentiation of human alveolar bone-derived mesenchymal stem cells (hABMSCs). ELF-PEMFs stimulation may be clinically beneficial in the treatment of fracture healing, especially in nonunions [1–3]. While there is a relatively frequent clinical use of electromagnetic stimulation, current evidence is insufficient to conclude a benefit of this treatment modality [4]. Aaron and Ciombor suggested that ELF-PEMFs-enhanced differentiation of mesenchymal stem cells is most likely responsible for the increase in extracellular matrix synthesis and bone maturation [5]. Recent studies indicated that progenitor cells might migrate into bone fracture sites and initiate osteogenic lineage commitment [6].

However, little is known about direct ELF-PEMFs-induced effects on osteoprogenitor cells as the most likely cell population contributing to the osteogenic response [6–9]. Only recently, Tsai et al. demonstrated a modulating role of ELF-PEMFs stimulation in MSC osteogenesis [8]. Furthermore, Sun et al. postulated that ELF-PEMFs exposure could enhance bone marrow mesenchymal stem cells proliferation [9]. To induce a biological response, translation of the electromagnetic signal into a biochemical signal is obligatory. Various, albeit somewhat conflicting, effects of ELF-PEMFs on transcriptional level and cell proliferation and differentiation have been reported in osteoblasts [10–16]. Multiple studies report positive effects of ELF-PEMFs on mineralization in osteoblast-like cell cultures [17–19]. Besides, ELF-PEMFs-induced effects on cellular differentiation, there is increasing evidence suggesting that the effects of electromagnetic stimulation are also dependent on cellular maturation stage [20, 21].

Diniz et al. showed that ELF-PEMF had a stimulatory effect on the osteoblasts in the early stages of culture which increased bone tissue-like formation but decreased bone tissue-like formation in the mineralization stage. Although many factors are known to be involved in bone growth and repair, the transforming growth factor beta (TGF-b1) family of proteins, including bone morphogenetic proteins (BMPs), are of particular interest due to their well-recognized osteogenic potential [22–24].

Although the results have been somewhat controversial, a variety of cell responses have been observed involving proliferation and differentiation [25], gene expression [26], modulation of the membrane receptors functionality [27], apoptosis [28], alteration in ion homeostasis [28, 29], and free radicals generation [30–33]. We thought that ELF-PEMFs directly could stimulate osteoprogenitors towards osteogenic differentiation, and the paper showed the cell proliferation and osteogenic differentiation effects of ELF-PEMFs on hABMSCs. To date, there have been no studies investigating the effects of the pulsed electromagnetic field stimulation treatments on tooth tissue approaches.

Ultimately, the purpose of the research was to develop an optimized and appropriately characterized noninvasive treatment via ELF-PEMFs. Thus, the paper described preliminary findings regarding the effect of ELF-PEMFs exposure specifically on the proliferation and differentiation of hABMSCs.

## 2. Materials and Methods

### 2.1. Cell Culture

The cells were collected at the Intellectual Biointerface Engineering Center, Dental Research Institute, College of Dentistry, Seoul National University. Cells were cultured in *α*-minimum essential medium (MEM) containing 10% fetal bovine serum (FBS, Welgene Inc., Republic of Korea), 10 mM ascorbic acid (L-ascorbic acid), antibiotics, and sodium bicarbonate at 37°C in a humidified atmosphere of 5% CO_2_ (Steri-Cycle 370 Incubator, Thermo Fisher Scientific. USA). The medium was changed every other day. hABMSCs were cultured after 24 h to facilitate attachment. When the cells became confluent, they were detached with 1 mL trypsin-EDTA, counted, and passaged. The cells were passaged before reaching confluence and used at between five and six passages.

### 2.2. Induction of Extremely Low Frequency Pulsed Electromagnetic Fields and Experimental Devices

**Figure 1:**
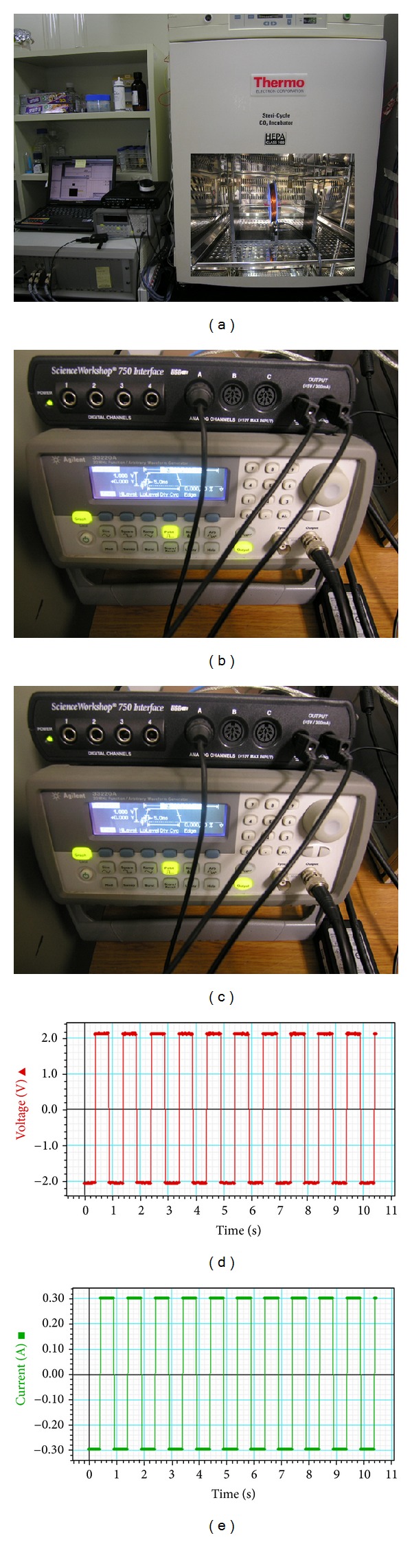
An extremely low frequency pulsed electromagnetic fields apparatus developed for this study; electromagnetic field apparatus system and CO_2_ incubator (a) the magnetic field sensor connected in conjunction with a channel of the ScienceWorkshop 750 USB interface (b). Electromagnetic field apparatus and the electromagnetic field exposure system put in an incubator in which the culture dish was placed in the core of the solenoid. The magnetic flux density was monitored via the PASCO's DataStudio software to collect and analyze data (c–e).

**Figure 2:**
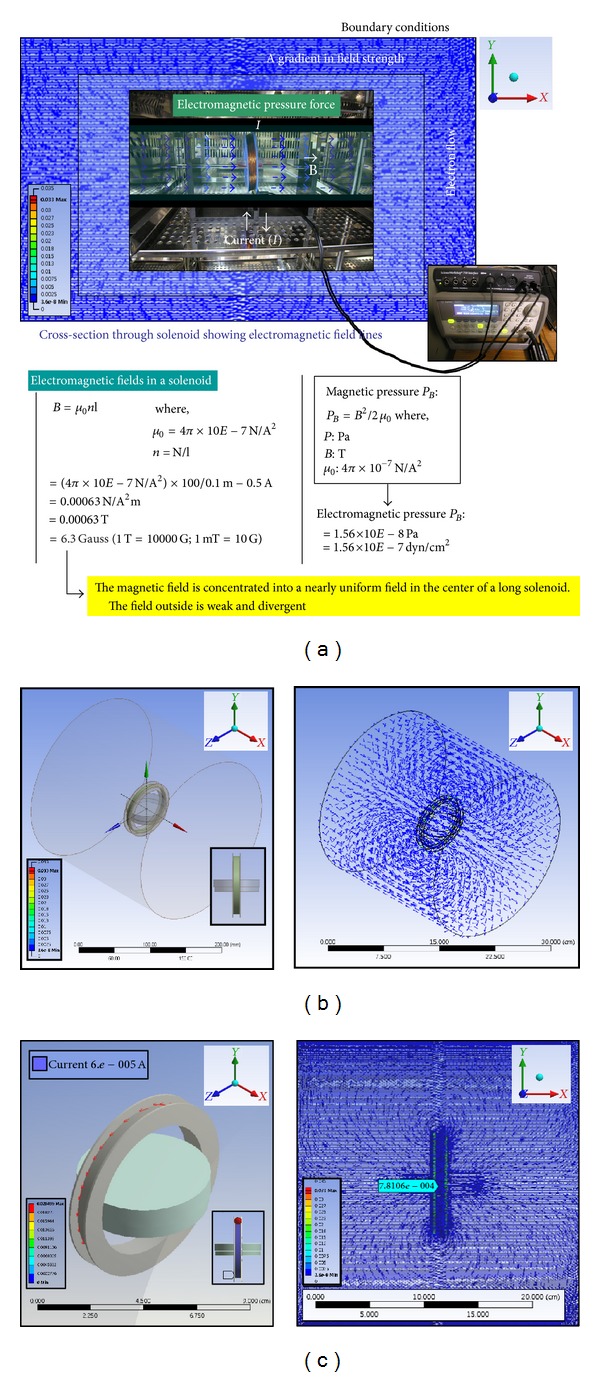
Electromagnetic fields and modeling by CFD. The electromagnetic pressure force is an energy density associated with the magnetic field strength by electric fields. We calculated electromagnetic fields values (a) in a solenoid which was concentrated into nearly uniform fields (b) in the center of a long solenoid (c).

The ELF-PEMFs stimulation system was developed for this study that ensures relatively uniform electromagnetic fields for cell culture. This apparatus included a waveform generator from 0 to 5 V and coils. The approximate average flux densities of the magnetic fields produced within the exposure apparatus of electromagnetic field was about 6 G ± 0.5. The cells were continuously exposed to 10, 30, and 100 Hz ELF-PEMFs, respectively. The magnetic field sensor (CI-6520A, PASCO Ltd., CA, USA) is used in conjunction with a channel of the ScienceWorkshop 750 USB interface (CI-7599, PASCO Ltd., CA, USA) as shown in Figure 1. The magnetic flux density was monitored with a Gauss meter at the center of the top of each culture dishes, where hABMSCs attached themselves to the culture dishes. The culture dishes of the control group were placed next to those of experimental group in the same CO_2_ incubator. The flux density values of the culture dishes were no greater than 0.05 mT, the level of the natural magnetic field of the earth. The magnetic flux density was monitored via the PASCO's DataStudio software (CI-7599, PASCO Ltd, CA, USA) to collect and analyze data. Exposure was performed in the air gap of the iron core with the magnetic field verticality plane to the cell cultures. Exposure apparatus of ELF-PEMFs prepared for our study and 60 mm in diameter culture dish (Nunc, Denmark) were used. The electromagnetic field exposure system was put in an incubator at 37°C; the culture dish was placed in the core of the solenoid where a homogeneous pulsed magnetic fields were generated (Figure 2), while control group was placed in a separate incubator.

### 2.3. Cell Proliferation and Viability Test

hABMSCs proliferation was measured by WST-1 assay (EZ-Cytox cell viability assay kit, Daeillab Service Co., LTD) as manufacture's protocols. The formazan dye produced by viable cells was quantified by a multiwell spectrophotometer (Victor 3, Perkin Elmer, USA), measuring the absorbance of the dye solution at 460 nm. DNA concentration was quantified by fluorometry using the CyQUANT cell proliferation assay kit (Invitrogen) and the *λ* DNA standard (Invitrogen) as manufacture's protocols. The CyQUANT is based on a strong increase in fluorescence of the CyQUANT GR dye when it binds cellular nucleic acids. Fluorescence was measured using a Cytofluor II fluorescence multiwell plate reader with excitation of 485 nm and emission of 530 nm. Statistical significance was analyzed between the nontreatment and ELF-PEMFs groups. Values of *P < 0.05 were considered to be statistically significant.

### 2.4. *In Vitro* Migration Study

hABMSCs were cultured with or without ELF-PEMFs, and cell morphology was observed by phase contrast microscopy (Nikon TS100, Japan). *In vitro* cell migration was assessed by CytoSelect Wound Healing Assay as manufacture's protocols. Wound closure was measured by microscopy for up to 72 hours, and photographs were taken. Cells were cultured with or without ELF-PEMFS, and cell morphology was periodically observed by phase contrast microscopy (Nikon TS100, Japan). hABMSCs were stimulated with exposure to ELF-PEMFs for 72 hours except for the control (without stimulation group). 

### 2.5. Measurement of Alkaline Phosphatase Activity

Alkaline phosphatase (ALP) activity of the cell layer was quantified spectrophotometrically according to the instructions of the SensoLyteTM ALP Assay kit (AnaApec, USA). After centrifugation at 2500 ×g for 10 min at 4°C, enzyme activity was calculated by measuring the yellow p-nitrophenol product formed at 405 nm (Victor 3, Perkin Elmer, USA).

### 2.6. Measurement of Mineralized Nodule Formation

hABMSCs were placed in 35 mm culture dishes at a density of 1.0 × 10^4^ cells/cm^2^ and cultured for about 2 weeks in *α*-MEM containing 50 mM *β*-glycerophosphate and 50 *μ*g/mL ascorbic acid, with and without ELF-PEMFs. The induction culture medium was changed every second or third day. The cells were exposed to ELF-PEMFs for 2 weeks (10 min duration/day) except for control (without stimulation group). Condition and nodule formation were checked routinely by phase contrast microscopy. The presence of mineralized nodules (calcium deposition) was determined by staining with alizarin red, as described [34]. The ethanol-fixed cells and matrix were stained for 1 h with 40 mM alizarin red-S (pH 4.2) and extensively rinsed with water. After photography, the bound stain was eluted with 10% (wt/vol) cetylpyridinium chloride, and alizarin red-S in samples was quantified by measuring absorbance at 544 nm (Victor 3, Perkin Elmer, USA). Vitamin C, *β*-glycerophosphate, alizarin red-S, and cetylpyridinium chloride were obtained from Sigma-Aldrich (St. Louis, MO, USA). 

### 2.7. Fluorescence Microscopy Analysis

The cells were washed in phosphate buffered saline (PBS, Sigma-Aldrich, Milwaukee, WI, USA), fixed in a 4% paraformaldehyde solution (Sigma-Aldrich, Milwaukee, WI, USA) for 20 min, and permeabilized with 0.2% Triton X-100 (Sigma-Aldrich, WI, Milwaukee, USA) for 15 min. Cells were incubated with TRITC-conjugated phalloidin, anti-vinculin, its secondary antibody (Millipore Cat. no. AP124F), and 4, 6-diamidino-2-phrnykinodole (DAPI; Millipore, Billerica, MA, USA) for 1 h to stain actin filaments, focal contracts, and nuclei, respectively. Cytoskeleton organization was visualized using an actin cytoskeleton and focal adhesion staining kit (FAK100; Millipore, Billerica, MA) according to the manufacturer's instruction. In addition, stem cell surface markers of mesenchymal stem cells were captured using STRO-1 (Santa Cruz Biotechnology, USA) and CD146 (BD bioscience, USA) according to the manufacturer's instruction. Cells were mounted in glycerol/buffer on a glass slide after extensive washing with PBS. Images of labeled cells were obtained by a fluorescence image restoration microscope (Applied Precision, USA).

### 2.8. Confocal Microscopy and Immunohistochemistry

Cells were washed in phosphate buffered saline (PBS, Sigma-Aldrich, Milwaukee, WI, USA), fixed in a 4% paraformaldehyde solution (Sigma-Aldrich, Milwaukee, WI, USA) for 20 min, and permeabilized with 0.2% Triton X-100 (Sigma-Aldrich, WI, Milwaukee, USA) for 15 min. Cells were incubated with TRITC-conjugated Phalloidin, anti-osteocalcin, its secondary antibody (Cat. no. AB10911, millipore), and 4, 6-diamidino-2-phrnykinodole (DAPI; Millipore, Billerica, MA, USA) for 1 h to stain actin filaments, focal contracts, and nuclei, respectively. In addition, the major intermediate filament protein of mesenchymal stem cells was visualized using an anti-Connexins 43 (Cat. no. AB1728, Millpore) according to the manufacturer's instruction. Connexin 43 is a member of the connexin gene family and a component of gap junctions. Gap junctions are composed of arrays of intercellular channels and provide a route for the diffusion of materials of low molecular weight from cell to cell. Negative controls were used during immunostaining by omitting primary antibodies, and at least two independent stainings were performed. Cells were mounted in glycerol/buffer on a glass slide after extensive washing with PBS. Images of labeled cells were obtained by a confocal laser scanning microscope (Carl Zeiss, LSM710).

### 2.9. Statistical Analysis

Statistical analysis was carried out using the statistical analysis system (SAS) for Windows v8.2 (SAS Institute, Inc., Cary, NC, USA). Statistical significance between control and treatment groups was compared with two-way ANOVA and Duncan's multiple range tests at *P* < 0.05. The data were reported as the mean ± standard deviation.

## 3. Results and Discussion

### 3.1. Cell Morphology, Cell Viability, and Proliferation Enhanced by ELF-PEMFs Induction

**Figure 3:**
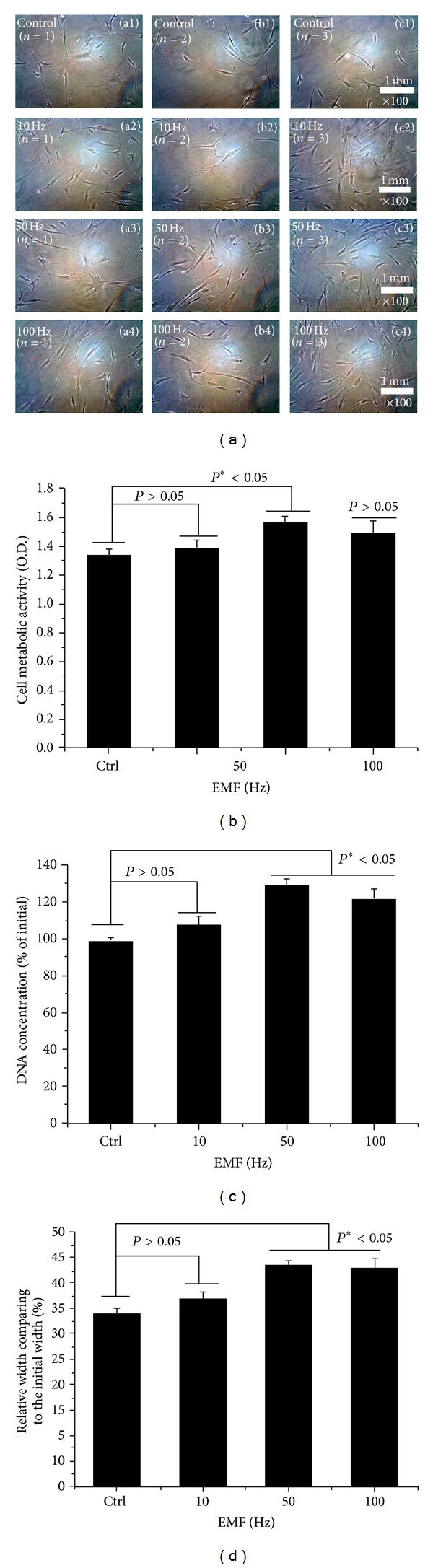
Representative optical microscopic images (a, *n* = 3) of hABMSCs exposed for 4 days in static condition (a1–c1) or ELF-PEMFs (intensity, 6 Gauss) induction at 10 Hz/day (a2–c2), 50 Hz/day (a3–c3), and 100 Hz/day. Cell metabolic viabilities as optical density (O.D.) of hABMSCs measured using WST-1 (b). DNA concentration (c) as a percent of initial of hABMSCs measured using CyQUANT cell proliferation assay kit (*n* = 3). *In vitro* cell migration indicating that ELF-PEMFs (intensity, 6 Gauss) group exposed at 10, 50, and 100 Hz in 6 G. Fifty and 100 Hz were statistically significant differences (*P < 0.05) among groups (d) (*n* = 3). Overhead brackets with asterisks indicate statistically significant differences between groups.

Cell morphologies were shown in representative optical microscopic images (Figure 3(a), *n* = 3) of hABMSCs stimulated by ELF-PEMFs induction for 4 days in one of the following exposure conditions: static condition (a1–c1) or ELF-PEMFs (intensity, 6 Gauss) induction at 10 Hz/day (a2–c2), 50 Hz/day (a3–c3), and 100 Hz/day. Cells showed that ELF-PEMFs induction-stimulated groups had greater cell numbers and cell growth than the static group. Cell metabolic viabilities were measured as optical densities of hABMSCs using WST-1 (Figure 3(b)). DNA concentration (Figure 3(c)) as the percent of initial of hABMSCs measured using CyQUANT cell proliferation assay kit (*n* = 3). *In vitro* cell migration as representative optical microscopic images with ELF-PEMFs induction groups compared to static culture (Figure 3(d)), indicating that ELF-PEMFs (intensity, 6 Gauss) group exposed at 10, 50, and 100 Hz in 6 G. Fifty and 100 Hz were statistically significant differences (*P < 0.05) among groups (d) (*n* = 3). Overhead brackets with asterisks indicate statistically significant differences between groups. The growth of hABMSCs was significantly increased at the ELF-PEMFs intensity levels, indicating that they are the optimal conditions. ELF-PEMFs have previously been associated with increased collagen deposition, enhanced ion transport and amino acid uptake, fibroblast migration, and ATP and protein synthesis. ELF-PEMFs may have an effect on intracellular ion control, especially Ca^2+^, as well as on mRNA expression, protein synthesis, and gene expression [35]. Consequently, we observed that lower ELF-PEMFs intensities (intensity, 6 Gauss) and wavelength (optimal frequency) induced greater cell metabolic activity. The hABMSCs proliferated and expanded significantly better under ELF-PEMFs of 50 and 100 Hz groups than in the control.

### 3.2. Synergistic Effects of Osteogenic Differentiation by ELF-PEMFs Induction

**Figure 4:**
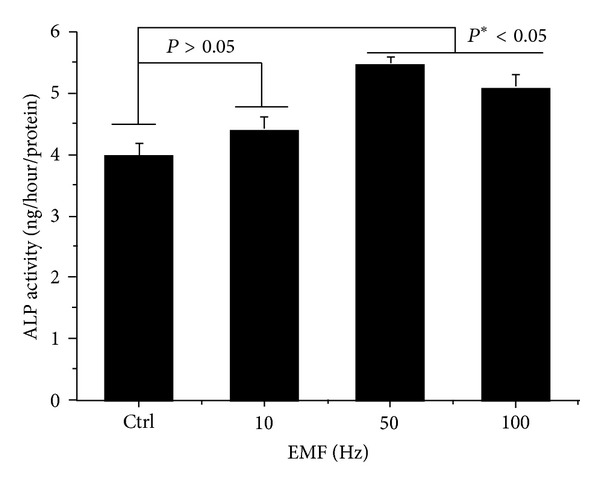
ALP activity cultured in different types of hABMSCs exposed with ELF-PEMFs (intensity, 6 Gauss) for 7 days. ELF-PEMFs induction group exposed at 50 mT/day and 100 mT/day (*P < 0.05) have statistically significant differences among groups (*n* = 3).

**Figure 5:**
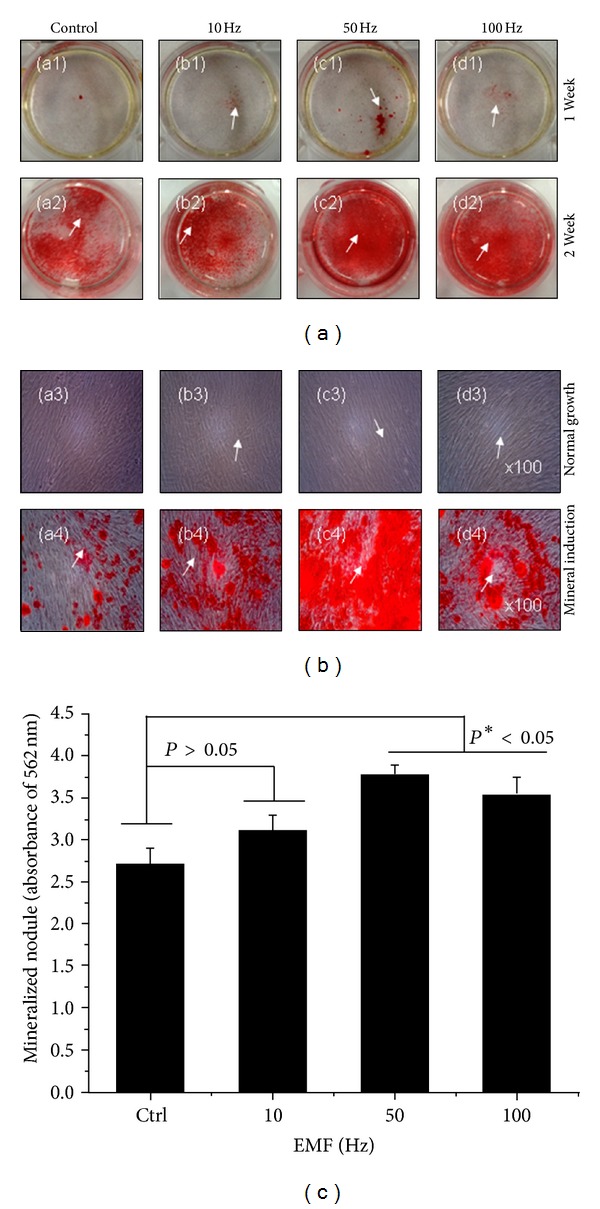
Representative optical microscopic images of hABMSCs after alizarin red staining treatment with static condition (a1, a2) or ELF-PEMFs (intensity, 6 Gauss) at 10 Hz/day (b1, b2), 50 Hz/day (c1, c2), and 100 Hz/day (d1, d2) at 1 and 2 weeks, respectively. ELF-PEMFs induction groups at 50 and 100 Hz/day were way too much intense compared to control (b, white arrows: mineral nodules stained in red). Mineralized nodule as optical density (absorbance of 562 nm) measured after destaining treatment (c). ELF-PEMFs induction exposed at 50 and 100 Hz/day groups have statistically significant differences (*P < 0.05) among groups (*n* = 3, bar = 1 mm).

ALP activity was maintained during the 7 days of culture (Figure 4). ELF-PEMFs groups exposed at 50 and 100 Hz/day (*P < 0.05) had statistically significant group differences. The early osteoblastic marker was also expressed over the range of ELF-PEMFs. We also investigated the effects of long-term ELF-PEMFs on the differentiation of hABMSCs. We observed that the formation of mineralized nodules is one of the markers of osteoblastic maturation.  Figure 5(a) showed representative optical microscopic images of hABMSCs after alizarin red staining treatment with static condition (a1, a2) or ELF-PEMFs (intensity, 6 Gauss) at 10 Hz/day (b1, b2), 50 Hz/day (c1, c2), and 100 Hz/day (d1, d2) at 1 and 2 weeks, respectively. ELF-PEMFs induction groups at 50 and 100 Hz/day were way too much intense compared to control (Figure 5(b), white arrows: mineral nodules stained in red). Mineralized nodule as optical density (absorbance of 562 nm) measured after destaining treatment Figure 5(c). ELF-PEMFs induction exposed at 50 and 100 Hz/day groups was statistically significant differences (*P < 0.05) among groups. (*n* = 3, bar = 1 mm). The hABMSCs cultured with ELF-PEMFs under conditioned media showed increased calcium contents, whereas the cells cultured under normal growth media showed no or a low increase of calcium, despite ELF-PEMF treatment (Figure 5(b): a3–d3).Figure 5(c) showed the optical density value of mineralized nodules (absorbance of 562 nm) measured after destaining treatment. ELF-PEMFs induction groups had statistically significant differences (*P < 0.05, 50 and 100 Hz/day; *n* = 3, bar = 1 mm). These results showed that optimal ELF-PEMFs induction with the proper intensity and frequency condition could enhance the differentiation and maturation of hABMSCs synergistically.

### 3.3. Increased Expression of CaM via ELF-PEMFs

**Figure 6:**
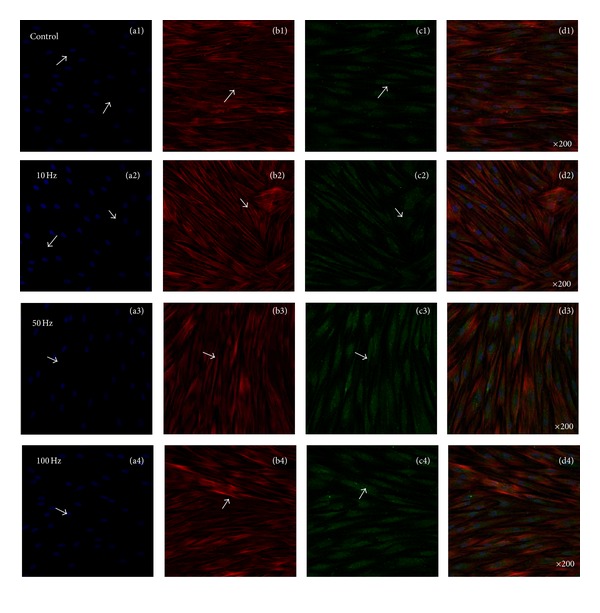
Representative confocal laser microscopy images of hABMSCs cultured for 7 days in static conditions (a1–d1) or ELF-PEMFs induction (intensity, 6 Gauss) at 10 Hz/day (a2–d2), 50 Hz/day (a3–d3), and 100 Hz/day (a4–d4) groups; cell nuclei (a1–d4), actin filaments (b1–b4), calmodulin (CaM, c1–c4), and merged images (d1–d4) of the fluorescence stains. Confocal laser microscopy images showed more intense observation at ELF-PEMFs induction groups compared to control group (arrows: cell direction).

Figure 6 showed representative confocal laser microscopy images of hABMSCs cultured for 7 days in static conditions (a1–d1) or ELF-PEMFs induction (intensity, 6 Gauss) at 10 Hz/day (a2–d2), 50 Hz/day (a3–d3), and 100 Hz/day (a4–d4) groups; cell nuclei (a1–d4), actin filaments (b1–b4), vimentin (c1–c4), and merged images (d1–d4) of the fluorescence stains. Confocal laser microscopy images showed more intense observation at ELF-PEMFs induction groups compared to control group (arrows: cell direction). The role of calcium ions (Ca^2+^) in cell function is beginning to be unraveled at the molecular level as a result of recent research on calcium-binding proteins and particularly on CaM. Calcium, in conjunction with the calcium-binding protein CaM, is a key mediator in signal transduction [36].

For external stimuli such as ELF-PEMFs to affect the behavior of proliferation and differentiation of hABMSCs, signal transduction must occur across the cell membrane. There are two approaches to signal transduction: activation of transmembrane channels or alteration of transmembrane receptors. Preliminary observations made by Aaron et al. suggested that the cellular response to ELF-PEMFs exposure might involve the calcium/CaM pathway [37]. However, inconsistent results regarding transmembrane channel activation still exist among researchers. On the other hand, the effectiveness of ELF-PEMFs may depend on a series of amplification mechanisms that occur during transmembrane coupling. The likely sites of amplification are the transmembrane receptors. Studies have shown that ELF-PEMFs alter membrane functions such as ion channels, ligand binding, and alterations in the density and distribution of receptors [38–40]. Each of these mechanisms has the ability to affect transmembrane signaling. As reported, hABMSCs growth was a significant difference (*P < 0.05) with respect to the control. These findings indicated that ELF-PEMFs for hABMSCs had the ability to enhance cell proliferation and differentiation as one of the noninvasive stimulation methods.

### 3.4. Enhanced Adhesion of Vinculin and Vimentin via ELF-PEMFs

**Figure 7:**
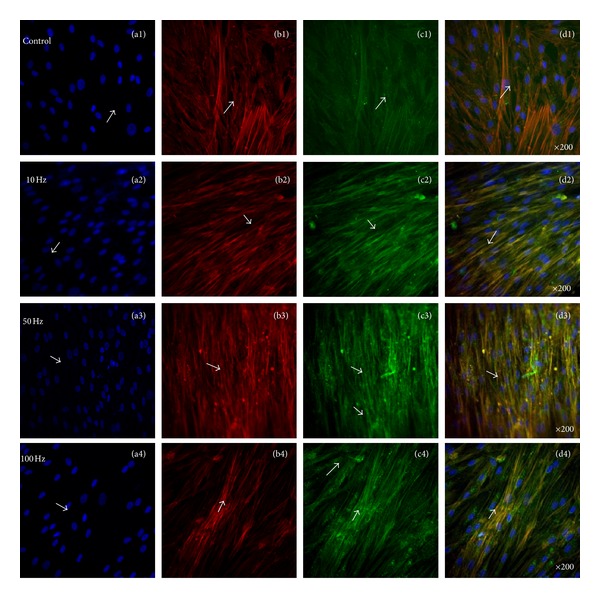
Representative confocal laser microscopy images of hABMSCs cultured for 7 days in static conditions (a1–d1) or ELF-PEMFs induction (intensity, 6 Gauss) at 10 Hz/day (a2–d2), 50 Hz/day (a3–d3), and 100 Hz/day (a4–d4) groups; cell nuclei (a1–d4), actin filaments (b1–b4), vinculin (c1–c4), and merged images (d1–d4) of the fluorescence stains. Confocal laser microscopy images showed more intense observation at ELF-PEMFs induction groups compared to control group (arrows: cell direction).

**Figure 8:**
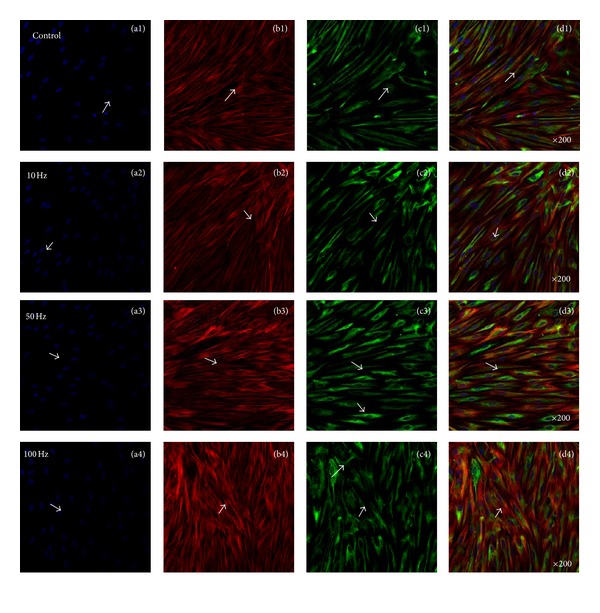
Representative confocal laser microscopy images of hABMSCs cultured for 7 days in static conditions (a1–d1) or ELF-PEMFs induction (intensity, 6 Gauss) at 10 Hz/day (a2–d2), 50 Hz/day (a3–d3), and 100 Hz/day (a4–d4) groups; cell nuclei (a1–d4), actin filaments (b1–b4), vimentin (c1–c4), and merged images (d1–d4) of the fluorescence stains. Confocal laser microscopy images showed more intense observation at ELF-PEMFs induction groups compared to control group (arrows: cell direction).

Figure 7 showed representative confocal laser microscopy images of hABMSCs cultured for 7 days in static conditions (a1–d1) or ELF-PEMFs induction (intensity, 6 Gauss) at 10 Hz/day (a2–d2), 50 Hz/day (a3–d3), and 100 Hz/day (a4–d4) groups; cell nuclei (a1–d4), actin filaments (b1–b4), vinculin (c1–c4), and merged images (d1–d4) of the fluorescence stains. Confocal laser microscopy images showed more intense observation at ELF-PEMFs induction groups compared to control group (arrows: cell direction).  Figure 8 showed representative confocal laser microscopy images of hABMSCs cultured for 7 days in static conditions (a1–d1) or ELF-PEMFs induction (intensity, 6 Gauss) at 10 Hz/day (a2–d2), 50 Hz/day (a3–d3), and 100 Hz/day (a4–d4) groups; cell nuclei (a1–d4), actin filaments (b1–b4), vimentin (c1–c4), and merged images (d1–d4) of the fluorescence stains. Confocal laser microscopy images showed more intense observation at ELF-PEMFs induction groups compared to control group (arrows: cell direction). Recent studies have revealed that also vimentin has key roles in adhesion by regulating integrin functions. Among the large protein family of intermediate filaments, vimentin is one of the most familiar members, as it is the major intermediate filaments (IFs) protein in mesenchymal cells, and it is frequently used as a developmental marker of cells and tissues. It is well established that IFs have an important role in adhesion and cell-cell interactions through their association with hemidesmosomes and desmosomes [41]. Recent studies have revealed that also vimentin has key roles in adhesion by regulating integrin functions.

### 3.5. Enhanced Osteoinduction of Osteocalcin via ELF-PEMFs

**Figure 9:**
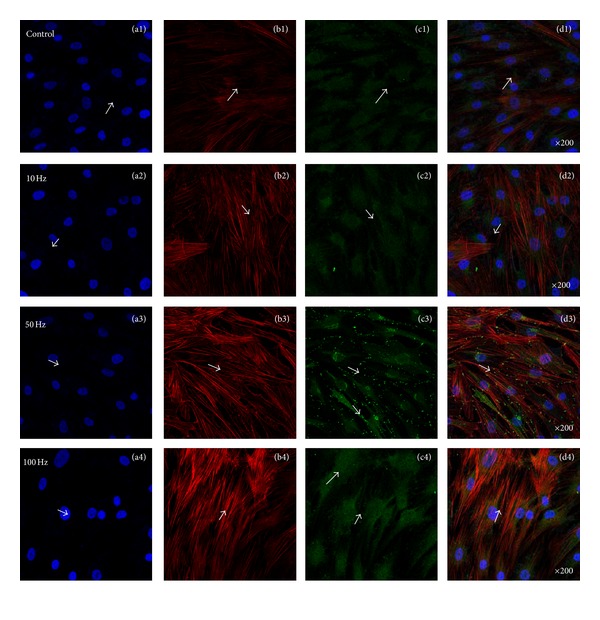
Representative confocal laser microscopy images of hABMSCs cultured for 7 days in static conditions (a1–d1) or ELF-PEMFs induction (intensity, 6 Gauss) at 10 Hz/day (a2–d2), 50 Hz/day (a3–d3), and 100 Hz/day (a4–d4) groups; cell nuclei (a1–d4), actin filaments (b1–b4), osteocalcin (OCN, c1–c4), and merged images (d1–d4) of the fluorescence stains. Confocal laser microscopy images showed more intense observation at ELF-PEMFs induction groups compared to control group (arrows: cell direction).

Figure 9 showed representative confocal laser microscopy images of hABMSCs cultured for 7 days in static conditions (a1–d1) or ELF-PEMFs induction (intensity, 6 Gauss) at 10 Hz/day (a2–d2), 50 Hz/day (a3–d3), and 100 Hz/day (a4–d4) groups; cell nuclei (a1–d4), actin filaments (b1–b4), osteocalcin (OCN, c1–c4), and merged images (d1–d4) of the fluorescence stains. Confocal laser microscopy images showed more intense observation at ELF-PEMFs induction groups compared to control group (arrows: cell direction). The CaM could promote the proliferation and differentiation of hABMSCs via the effect of altered Ca^2+^ concentrations which could provide evidence for CaM signaling in cellular migration, proliferation, and differentiation. Thus, signal transduction via ELF-PEMFs ultimately could affect enhanced adhesion molecules and then finally promote enhanced osteogenesis. The results suggest that ELF-PEMFs at the proper intensity enhance the differentiation and maturation of hABMSCs.

The influence of ELF-PEMFs on cell proliferation in the mT range of magnetic flux density has been investigated by many authors. Khalil and Qassem [42] noted a decrease in proliferation index after exposure of lymphocytes to a 1 mT 50 Hz field. A single exposure for 60 min to a 2 mT 50 Hz magnetic field led to a decrease in cell number of about −10% of control SV40-3T3 monolayers 6 h subsequent to the exposure [43]. These biological factors are known to be strong modifiers of the cellular response towards the fields [44–46]. The cell membrane has high impedance [47]. As a result, electric fields can polarize membrane components, move receptors or channels by electrophoresis within the membrane, or alter receptor conformation [48]. A past study has shown that DC electric fields can affect the assembly and distribution of actin filaments within the cytoplasm of endothelial cells [49, 50]. 

According to the electrochemical information transfer hypothesis, low level electromagnetic and permanent magnetic fields interact with cell membranes by enhancing the binding rate of ions with enzymes and receptors [51–53]. Ions such as calcium play an important role in regulating cell shape. Calcium is responsible for regulating changes in the actin filament meshwork that is present in the cytoplasm of chondrocytes [54]. According to the present mechanism, there is also an explanation why pulsed electromagnetic fields can be more bioactive than continuous fields of the same characteristics, or why the greatest effects of a continuous field may occur with onset or removal of exposure to this. Such phenomena have been observed in several experiments [55–57] and until now there was not any theoretical explanation.

Finally, our results that ELF-PEMFs induction is quite efficient for improving the proliferation and differentiation of hABMSCs suggest that it may be a good strategy for hABMSCs-based tissue engineering applications with suitable scaffolds, especially fabricated by conducting biomaterials [58–61].

## 4. Conclusions

This study was performed to investigate the effects of ELF-PEMFs induction on the proliferation and differentiation of hABMSCs. We studied cell proliferation, migration, mineralized nodule formation, von Kossa, and ALP activity as indicators of osteogenesis. The results indicated that ELF-PEMFs induction created an important synergistic effect for activating mechanotransduction. We found synergistic effects of ELF-PEMFs on the proliferation and differentiation of hABMSCs. Alizarin red staining showed that mineralized nodules formed intensely (intensity, 6 Gauss, frequency, 50 and 100 Hz). ALP activity in similar intensity groups had statistically significant group differences. We also examined vinculin, vimentin, and CaM expressions during osteogenic differentiation. ELF-PEMFs increased vinculin, vimentin, and CaM expressions, compared to control. In particular, CaM indicated that ELF-PEMFs significantly altered expression of osteogenesis-related genes. The results showed that ELF-PEMFs could enhance cell proliferation and accelerate the osteogenesis. In conclusion, the present findings could suggest that ELF-PEMFs at the proper intensity enhanced bone formation by promoting the differentiation and maturation of the stem cells.
